# Shining a light on *Candida*-induced epithelial damage with a luciferase reporter

**DOI:** 10.1128/msphere.00509-24

**Published:** 2024-10-16

**Authors:** Millen Tesfamariam, Raghav Vij, Verena Trümper, Bernhard Hube, Sascha Brunke

**Affiliations:** 1 Department of Molecular Pathogenicity Mechanisms, Leibniz Institute for Natural Product Research and Infection Biology-Hans Knoell Institute, Jena, Germany; 2 Institute of Microbiology, Friedrich Schiller University, Jena, Germany; University of Guelph, Guelph, Ontario, Canada

**Keywords:** mammalian damage, fungal pathogen, high-throughput screening, luciferase

## Abstract

Host cell damage is a key parameter for research in infection biology, drug testing, and substance safety screening. In this study, we introduce a luciferase reporter system as a new and reliable assay to measure cell damage and validate it with the pathogenic yeast, *Candida albicans*, as a test case. We transduced human epithelial cell lines with a lentiviral vector to stably express an optimized luciferase enzyme, Nanoluc. Upon cell damage, the release of cytoplasmic luciferase into the extracellular space can be easily detected by a luminometer. We used the luciferase reporter system to investigate the damage caused by *C. albicans* to different newly generated epithelial reporter cell lines. We found that fungus-induced cell damage, as determined by established methods, correlated tightly with the release of the luciferase. The new luciferase reporter system is a simple, sensitive, robust, and inexpensive method for measuring host cell damage and has a sensitivity comparable to the standard assay, release of lactate dehydrogenase. It is suitable for high-throughput studies of pathogenesis mechanisms of any microbe, for antimicrobial drug screening, and many other applications.

**IMPORTANCE:**

We present a quick, easy, inexpensive, and reliable assay to measure damage to mammalian cells. To this end, we created reporter cell lines which artificially express luciferase, an enzyme that can be easily detected in the supernatant when these cells are damaged. We used infections with the well-investigated fungal pathogen of humans, *Candida albicans*, as a test case of our system. Using our reporter, we were able to recapitulate the known effects of strain variability, gene deletions, and antifungal treatments on host cell damage. This easily adaptable reporter system can be used to screen for damage in infection models with different microbial species, assay cell-damaging potential of substances, discover new non-toxic antibiotics, and many other damage-based applications.

## INTRODUCTION

There are many reasons microorganisms damage host cells, e.g., to acquire nutrients for growth, to invade and colonize different niches, and to protect themselves against immune cells. Thus, infection biologists strive to understand the microbial virulence programs that cause damage to host cells and discover drugs that mitigate this damage. This necessitates easy, reliable, and accurate readouts for cytotoxicity or cell damage in *in vitro* assays. Many popular high-throughput measures of cytotoxicity (reviewed in references 1, 2) rely on the enzyme activity of cellular components released when host cells die, like lactate dehydrogenase (LDH), adenylate kinase or glyceraldehyde-3-phosphate dehydrogenase. However, these enzymes can also be found in many bacteria, fungi, and parasites, confounding cytotoxicity measurements in infection models. The enzyme assays also generally require multiple wash steps and often expensive and toxic buffers and substrates. In this manuscript, we introduce a high-throughput and inexpensive Nano-luciferase (Nanoluc or Nluc, Promega) reporter for cytotoxicity and examine its use and reliability in *Candida albicans* infection models as a study of potential applications.

Fungal diseases affect billions of people every year, yet there are only four classes of antifungals available to treat a wide range of fungal infections (3). *Candida* species are among the most common fungal pathogens and cause tens of millions of mucosal and nearly 700,000 invasive infections annually, with an unacceptable high morbidity and mortality (4). *Candida* sp. usually resides within humans as harmless commensals. From there, prolonged use of antibiotics or a weakened immune system can trigger mucosal or systemic candidiasis. *C. albicans*, the most prevalent and well-studied member of the clade, can cause deadly infections and is armed with virulence traits that help it survive and persist in the host. Among others, *C. albicans* adheres to host cells, invades and damages them, all while evading our immune responses (5). To investigate these virulence traits, readouts for host cell cytotoxicity (i.e., cell damage) have been indispensable. Methods like detection of LDH release (6, 7), release of radiolabeled chromium (^51^Cr) (8, 9), and fluorescence microscopy of propidium iodide (PI)-stained nuclei of dying cells (10), have all been used in the past with great success, for example, in the discovery of the fungal peptide toxin candidalysin (11). These assays come with specific drawbacks that hinder their application in high-throughput-screens: LDH cytotoxicity kits are comparatively expensive and inhibited by medium components like phenol red and pyruvate; quantifying fluorescence of nuclear cell-death dyes like PI is confounded by rapidly proliferating microbes in *in vitro* models that block the light signal; and both techniques are confounded by microbial LDH and nuclear staining. While ^51^Cr is highly sensitive and specific, it is also expensive and comes with the dangers and inconvenience of working with radioactivity. New high-throughput and inexpensive host cell cytotoxicity reporters can expedite the discovery of virulence factors and generally simplify cell damage assays.

In the 1880s, the French physiologist DuBois described the molecular basis of luminesce in fireflies, marine animals, and other organisms (12). The reaction of an enzyme called luciferase with its substrate luciferin leads to an emission of visible light (13). With advancements in molecular biology, scientists engineered ways to express luciferase in cells, and ever since, we have found a broad range of applications for luciferase-based assays to study protein expression and for high-throughput drug discovery screening (14–16).

**Fig 1:**
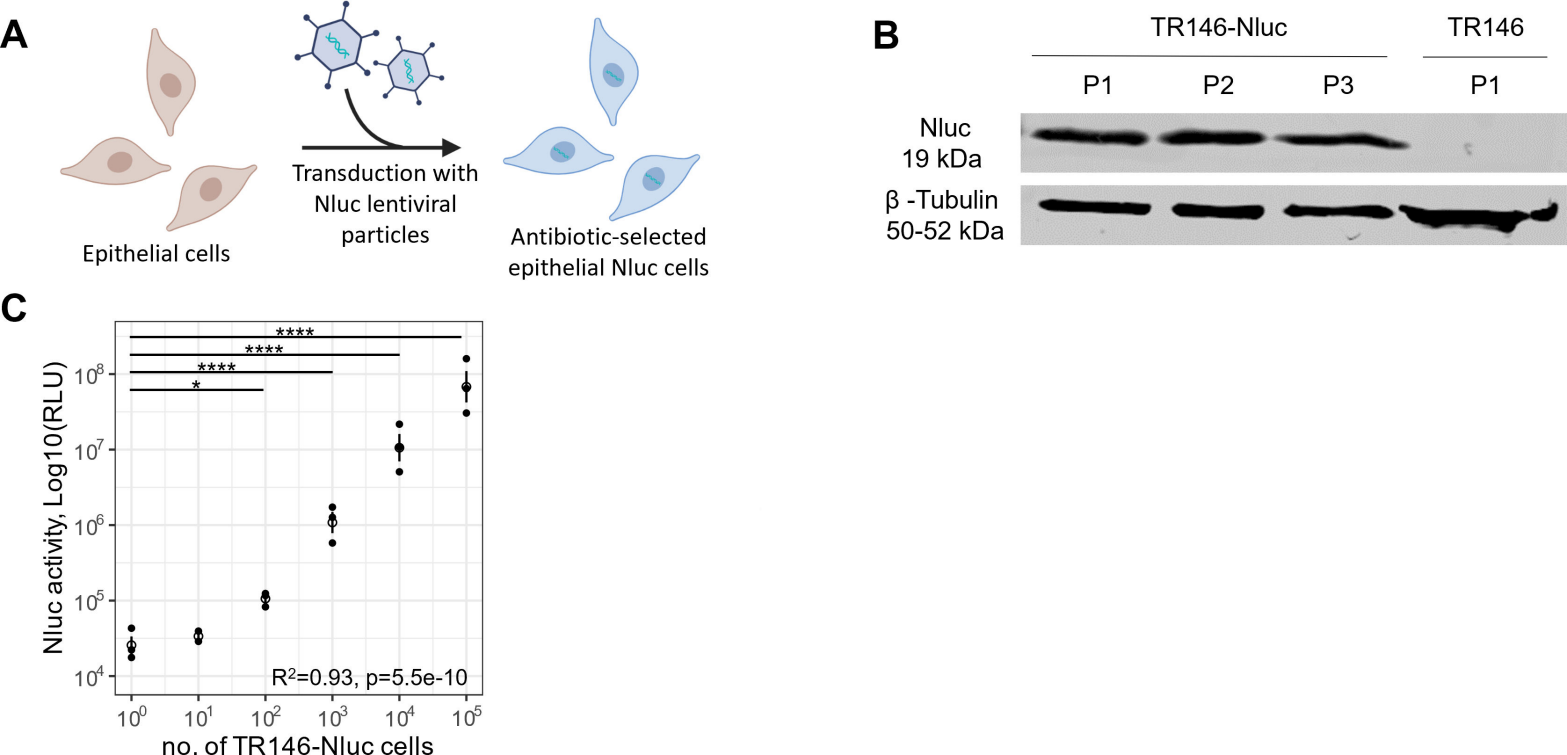
Nluc is stably expressed in transduced epithelial cells. (**A**) Schematic representation of lentiviral transduction to generate Nluc epithelial cell lines. The scheme was created using BioRender. (**B**) Cell lysates from TR146-Nluc were collected over three passages (P1-3), solubilized for SDS-PAGE, blotted and proteins were detected with the indicated antibodies. Representative image of three independent replicates shows that TR146-Nluc cells are stably expressing Nluc. (**C**) Luminescence of cell lysate correlated highly (*R*^2^ = 0.93) and significantly with the number of TR146-Nluc cells that were lysed. All experiments were performed in 2–3 independent replicates, where the white circles represent the value from each replicate. One-way ANOVA followed by Benjamini-Hochberg-corrected multiple comparison *t*-tests comparing each sample mean to the mean luciferase value from 10^0^ cell. Significance levels are represented by *, **, ***, and ****, indicating adjusted *P*-values of less than 0.05, 0.01, 0.001, and 0.0001, respectively.

In this manuscript, we designed and validated an epithelial damage reporter based on Nano-luciferase (Nluc) (17). We transduced oral, vaginal, and gut human epithelial cell lines (TR146 and A431) using a lentiviral vector to constitutively and stably express Nluc in the cytoplasm (Fig. 1A). With the underlying assumption that damaged epithelial cells “leak” proteins like Nluc and LDH into the extracellular space, we approximate host cell damage by assaying Nluc and LDH enzymatic activity in the supernatant. We show that using our Nluc assay we can track epithelial damage over the time course of *C. albicans* infections.

## RESULTS

### Luciferase activity from lysates of stably transduced epithelial Nluc cells correlates with the number of cells

We transduced TR146 and A431 cell lines with lentiviral particles containing a Nluc insert with a constitutively active promoter and a puromycin selection cassette. To confirm that TR146-Nluc cells stably expressed Nluc over time, we collected cells from different passages, lysed them, and performed a western blot with anti-Nluc antibody (Fig. 1B). The stable expression of Nluc in A431-Nluc was similarly confirmed by western blot (Fig. S1). We also noted that luciferase activity highly correlated with the number of cells lysed, and it reliably detected as few as 100 cells (Fig. 1C).

### Epithelial Nluc cell lines function as cytotoxicity reporters in *C. albicans* infection models

**Fig 2:**
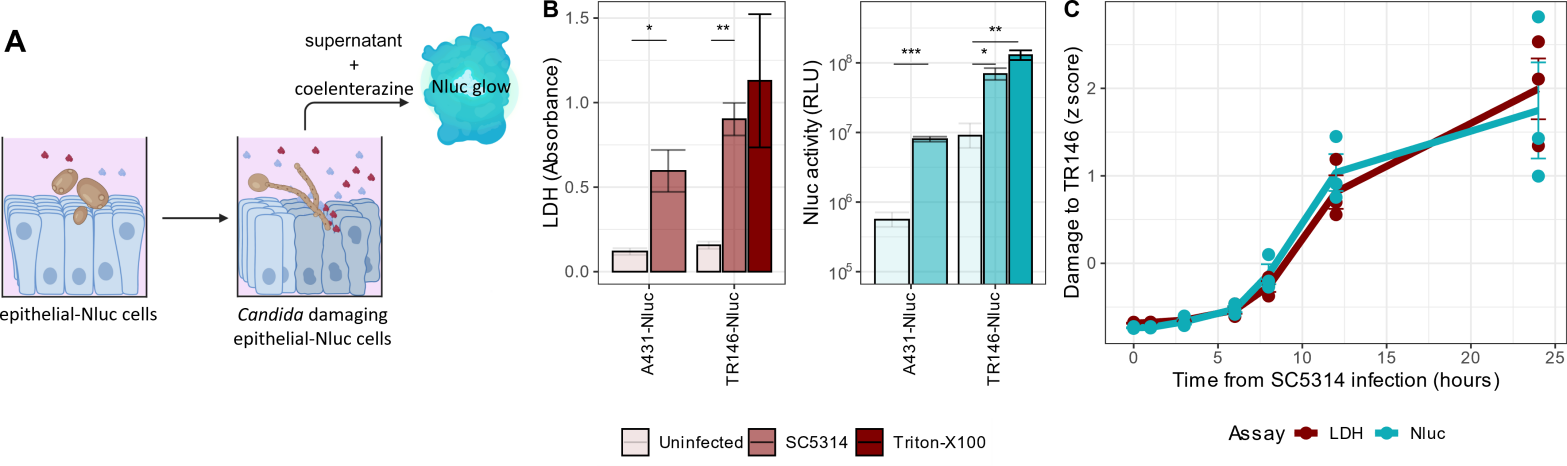
The Nluc luminescence assay reflects *C. albicans* (SC5314)-induced epithelial damage. (**A**) Scheme of Nluc-damage assay depicting transduced epithelial Nluc cells infected with *C. albicans. C. albicans* invades and damages epithelial cells, releasing cytoplasmic proteins Nluc (blue globules) and LDH (red globules) into the supernatant. The supernatant is collected and diluted for the assay. The luciferase substrate coelenterazine is added to the diluted supernatant, which leads to the emission of light that is measured by a luminometer. Scheme was created using BioRender. (**B**) Damage to Nluc-expressing vaginal (A431), or oral (TR146) epithelial cells infected with *C. albicans* was compared to uninfected cells, or cells lysed by Triton X-100, by measuring LDH and Nluc activity of supernatant collected 24 h after infection. Bars depict mean LDH or Nluc activity (log scaled), and error bars depict standard deviation of the mean. Samples were compared to uninfected controls by one-way ANOVA followed by Benjamini-Hochberg-corrected multiple comparison *t*-tests. Significance levels are represented by *, **, ***, **** denoting adjusted *P* value < 0.05, 0.01, 0.001, and 0.0001, respectively. All experiments were carried out in three independent replicates. (**C**) Damage to oral epithelial (TR146-Nluc) cells was estimated by Nluc (turquoise) and LDH (red) assays at different time points after infection with *C. albicans* (*x*-axis). Values on the *y*-axis are presented as *Z* scores, i.e., they are centered around their respective mean, to allow direct comparison of the two assays. Error bars represent the standard deviation of the mean. All experiments were carried out in three independent replicates.

We infected TR146-Nluc and A431-Nluc cells with *C. albicans* (genomic reference wild-type strain for *C. albicans*, SC5314) for 24 h and measured the Nluc activity in the supernatant. *C. albicans*-infected cells released significantly more luciferase than uninfected cells (Fig. 2B), showing that our system can detect fungal-induced epithelial cell damage. The Nluc cytotoxicity reporter assay can be performed simply by collecting 5–10 µL supernatant after spinning down the cell debris, diluting the supernatant to 100 µL with PBS, adding the luciferin, and immediately measuring the luminescence (Fig. 2A).

Next, we wanted to verify that the luciferase reporter cell line TR146-Nluc can recapitulate the temporal dynamics of a *C. albicans* infection as well as the popular cytotoxicity assay, LDH. To this end, we collected supernatants from infected TR146-Nluc cells at various timepoints up to 24 h and found that the readouts from LDH and luciferase assays were highly in agreement over the entire time course (Fig. 2C).

### Applications for Nluc-epithelial cytotoxicity reporter in *C. albicans in vitro* infection models

**Fig 3:**
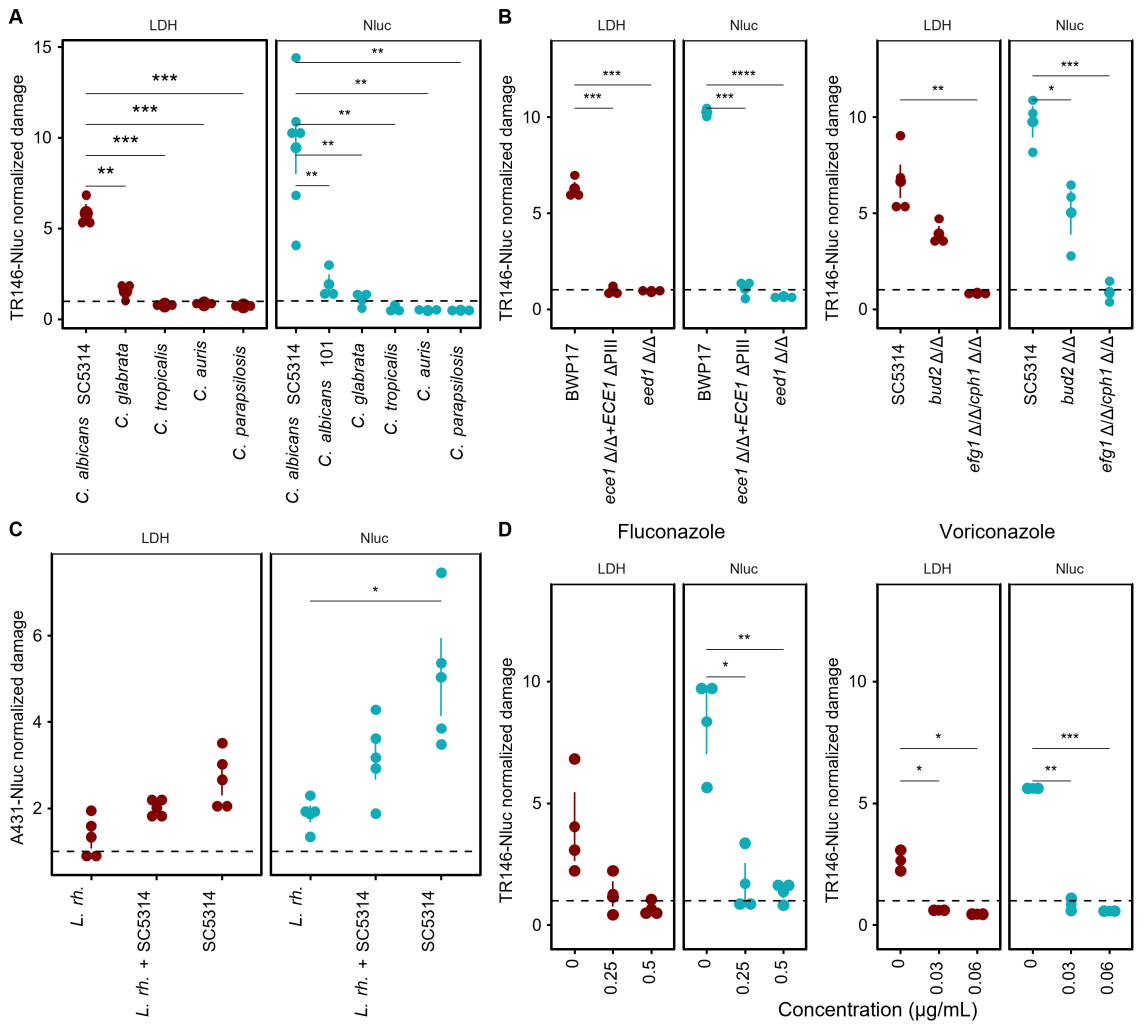
Applications for epithelial cells NLuc damage assays: (**A**) Damage to TR146-Nluc cells by different *Candida* species. (**B**) In both, LDH and Nluc assay *C. albicans* virulence factor deletion mutants show reduced damage to TR146-Nluc compared to their background strains BWP17 and SC5314. (**C**) In a commensal vaginal epithelial model, *L. rhamnosus* (*L. rh*.) protects A431-Nluc cells from damage against *C. albicans* infection. (**D**) Damage to TR146-Nluc cells infected with *C. albicans* (SC5314) at sub-MIC fluconazole and voricanazole concentrations. Damage to epithelial cells, estimated by LDH and Nluc assays, was normalized to uninfected controls (dotted line). Experiments were performed in two to three independent replicates, with three technical replicates each. The error bars represent the standard deviation of the mean. Means were compared to untreated controls by one-way ANOVA followed by Benjamini-Hochberg-corrected multiple comparison *t*-tests. Significance levels are represented by *, **, ***, and ****, indicating adjusted *P*-values of less than 0.05, 0.01, 0.001, and 0.0001, respectively.

Next, we tested the Nluc-based cytotoxicity reporter for typical applications in *C. albicans* infection biology and compared the luciferase readouts with the conventional LDH assay. As reported previously for such *in vitro* models, the filamenting *C. albicans* strain SC5314 caused significant damage to monolayers of oral, vaginal, and gut epithelial cells, but other *Candida* spp. including *C. glabrata*, *C. auris*, and *C. parapsilosis* did not elicit detectable host cell damage in LDH and Nluc assays (Fig. 3A). *C. albicans* strains can differ in their damage potential. For example, the *C. albicans* clinical isolate 101 is known to cause less damage in an oral epithelial infection model, as measured by LDH release, than the genome reference strain SC5314 (18). We were able to confirm this finding with our Nluc damage assay, showing that it is also useful for strain comparisons. *C. albicans* mutants with deletions for genes or sequences encoding the peptide toxin candidalysin (*ECE1-PIII*) (7), transcriptional regulators of filamentation (*EFG1/CPH1* or *EED1*) (19–21), or thigmotropism (*BUD2*) (22) are defective in virulence to different extents. They were attenuated in damaging TR146-Nluc cells (Fig. 3B), and the amount of damage reduction agreed well with the LDH assays done in parallel and with the phenotypes known from literature. Altogether, these experiments show that our epithelial-Nluc cell assay agrees well with the conventional and widely used LDH detection system and that it can be reliably used to screen and test different *Candida* species, *C. albicans* strains, and mutants.

Probiotics like *Lactobacillus rhamnosus* have recently garnered interest due to their ability to attenuate *C. albicans* virulence (23, 24). We asked if *L. rhamnosus* colonization can protect A431-Nluc cells from *C. albicans*-induced damage. We observed a clear trend toward reduced damage induced by *C. albicans* alone vs damage caused in combination with *L. rhamnosus* although these differences were not statistically significant (Fig. 3C). This potential reduction was seen in both assays, LDH and Nluc.

Next, we tested whether commonly used antifungals—fluconazole and voriconazole—can protect TR146-Nluc from *C. albicans-*induced cell damage and found that with Nluc, but not with LDH, we were able to reliably detect the protective effect of antifungals even at sub-MIC levels (Fig. 3D).

### Robustness of Nluc and LDH activity at different pH, temperature, and pyruvate concentrations

**Fig 4:**
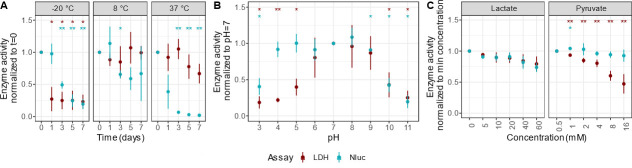
Limitations and strengths of the Nluc cytotoxicity assay: (**A**) Nluc and LDH activity of cell lysate incubated at −25°C, 8°C, and 37°C for 0–7 days. (**B**) Nluc and LDH activity of cell lysate incubated with PBS buffered at various pH values. (**C**) Nluc and LDH activity of cell lysates incubated with increasing concentrations of lactate and pyruvate. Experiments were performed in three independent replicates, with three technical replicates each. Error bars represent the standard deviation about the mean, where the mean is represented as a point. Means were compared by one-way ANOVA followed Benjamini-Hochberg-corrected *t*-tests against the normalized value of 1. Significance levels are represented by * and **, indicating adjusted *P*-values of less than 0.05 and 0.01, respectively. The color of the symbols shows the results of statistical comparisons for LDH and Nluc, respectively.

Supernatants from cell damage assays can be used immediately to measure cytotoxicity, or they can be stored for a while before measurement. To determine how long, and at what temperatures, Nluc and LDH activities are stable and accurate measures of cell damage, we stored TR146-Nluc cell lysate at −25°C, 8°C, and 37°C. We measured the Nluc and LDH activities from aliquots every other day over a week. The Nluc activity declined steeply on the third day of storing the supernatant at −25°C, while LDH activity was already mostly lost directly after freezing (Fig. 4A). Nluc and LDH activities remained mostly stable at 8°C. At 37°C, Nluc activity was lost over the first days, while LDH remained mostly stable for up to a week. Therefore, supernatants collected from epithelial-Nluc cells should be used to detect luciferase activity either immediately or stored at 8°C for up to a week or at −25°C for up to a day.

Infection of human cells often changes the pH of cell culture medium due to the metabolic activities of the microorganisms. We, therefore, tested the pH range at which Nluc (or LDH) activity is a reliable measure of cytotoxicity by diluting cell lysate in PBS buffered at different pH values and then assaying LDH and Nluc activity (25). We found that Nluc out-performed LDH and gave consistent readings from pH 4 to 9 (Fig. 4B), while the LDH assay was highly pH-dependent. Lastly, we examined the effect of the known LDH inhibitor pyruvate, which is also a common cell culture medium component, on Nluc, and found no discernible effect on Nluc enzyme activity (Fig. 4C).

## DISCUSSIONS

In this manuscript, we designed and optimized a luciferase reporter system to measure cytotoxicity (damage) in mammalian epithelial cell lines in an infection model. We used a lentiviral system to transduce immortalized epithelial cells taken from niches where invasive or superficial candidiasis typically occurs. This included the oral epithelial cell line TR146 and vaginal A431. With our simple luciferase assay, we were able to detect a measurable signal from as few as 100 TR146-Nluc cells. The Nluc cytotoxicity assays recapitulated the epithelial damage caused by *C. albicans* over the course of an infection, as observed by the popular LDH-based cytotoxicity assay. We also tested the application of TR146-Nluc to detect mammalian cytotoxicity by different *Candida* species, *C. albicans* mutants, and the protective effects of antifungals. In all assays, we found the Nluc data comparable and, in some cases, even superior to the LDH assay. Finally, we tested the stability of the Nluc and LDH enzymes in cell lysate over a range of temperatures, storage duration, and pH. The results of this study suggest that the luciferase-based assay is a promising new tool for the detection of epithelial cell damage caused by *C. albicans* infections.

The Nluc cytotoxicity assay detects even low levels of cell damage quickly, reliably, and inexpensively, making it ideal for high-throughput applications. After collecting the sample containing Nluc and diluting it in a simple buffer, the step of adding freshly prepared luciferin can be easily automated, and the resulting luminescence can be measured almost immediately. This contrasts with the time-consuming steps involved in measuring LDH activity from supernatants of samples, which requires the fresh preparation of a complex stabilizing buffer and a catalyst, adding this mixture to the diluted samples, waiting 15–30 min, and then measuring the absorbance. After the initial cost of transducing cell lines, the assay is inexpensive and determined mostly by the cost of coelenterazine, or other luciferase substrates. Luciferase-expressing cell lines created with non-proprietary luciferases (16) can further reduce the cost and be just as effective.

One limitation of using luminescence readouts is the lack of a protein standard to make standard curves, which correlate luminescence signal intensity to protein amount (ng/ mL) for each assay. Once the substrate luciferin is added, the reaction is instantaneous, and the luminescence signal decays over time. This leads to variability between independent replicates. Even with this limitation, in our manuscript, we demonstrated that the Nluc-reporter cell lines can clearly distinguish damaged vs non-damaged samples (Fig. 1B). There are also other ways to overcome the variability between assays. One is to use an automatized substrate dispenser to minimize the time between the addition of substrate and the measurement of luminescence. We can also create a calibration curve for each assay, by lysing a fixed number of cells, serially diluting them, and measuring the luminescence. As seen in Fig. 1C, the number of cells correlates very well with Nluc-luminescence signal, and this way, we were able to estimate the absolute number of cells damaged in each sample.

In our paper, we provided a proof-of-concept for our Nluc cytotoxicity reporter assay for a well-studied fungal pathogen, *C. albicans*. While non-*C*. *albicans* were conventionally thought to be “non-damaging” in epithelial cell-culture infection models (Fig. 3A), recently, it was discovered that adding albumin to the cell-culture medium increased *C. glabrata*-induced epithelial damage (26). Moving forward, the Nluc-assay can help us dissect the molecular mechanism of non-*C*. *albicans-*induced cell damage.

The extensive clinical use of antifungals to treat the increasing population at risk of *Candida* sp. infections, including people who are frequently hospitalized, the elderly, and the immunocompromised, has led to the emergence of drug resistance in *Candida* spp., particularly in *C. glabrata* (27). This, along with the urgent need to develop drugs to treat the emerging multi-drug-resistant *C. auris*, has led to calls for expedited antifungal drug discovery (reviewed in reference 28).

Among the many practical challenges in developing new antifungal drugs (29; reviewed in reference 30), a major one is the off-target toxicity of antifungal leads because both, infecting fungi and human host are eukaryotes, and many potential fungal targets are conserved between them. Most drug discovery efforts focus on first assaying antimicrobial activity in *in vitro* growth assays and then studying the side effects of the selected hits on human cells. We found that the Nluc-reporter cell line was sensitive in detecting protection from *C. albicans*-induced epithelial damage in the presence of subinhibitory levels of antifungals (Fig. 1D). This demonstrates a promising application for the Nluc-reporter cell lines that can be easily adapted to a one-step assay with simultaneous readouts for both mammalian cytotoxicity using the Nluc assay and antimicrobial effects using reporter microbe strains that quantify microbial biomass with microscopy (e.g., *Candida*-GFP). In this way, a host cell cytotoxicity reporter can expedite the discovery of non-toxic antifungal drugs and antivirulence drugs.

The public health risks have galvanized research and discovery in the *Candida* field, such that, now, *C. albicans* is a genetically tractable organism with large collections of gene knock-out libraries (31–33), well characterized *in vitro* models with a clear host-cell-damaging phenotype (34), and extensively studied antifungal drug interactions (35) (Fig. 3). The Nluc damage reporter can just as easily be applied to study damage to many other cell-types induced by any microorganism, drugs, noxious substances, and other host cells.

We also found that the lentiviral transduction system was easily adaptable for creating multiple Nluc-reporter cell lines. After engineering the appropriate plasmids and harvesting virus-like particles from transfected HEK-293T/17 cells, the viral particles could be stored at −80°C for an extended period and used to create and select new Nluc cell lines as needed, requiring minimal effort.

In this section, we will discuss several strengths of using our Nluc damage-reporter in place of the conventional LDH cytotoxicity assay. For instance, certain bacteria lower the pH of cell culture medium when co-cultured with human cells, and in these cases, LDH release assays underestimate mammalian-cell damage because the lowered pH inhibits LDH (25, 36). Given Nluc’s stability over a wide pH range [Fig. 4B (37)], Nluc-reporter cell lines will be particularly useful in assaying cell damage in such models and better mimic the low-pH physiologically relevant niches (38).

Furthermore, our Nluc-reporter assays damage specific to transduced cells. LDH, an enzyme found all across the kingdom of life, catalyzes pyruvate to lactate in many pathogens, including some fungi (39, 40), many bacteria (41), and protozoa (42, 43). Therefore, when developing infection models to study non-model organisms, it is prudent to use damage assays that do not rely on LDH. Because of these concerns, some groups have relied on ^51^Cr release to measure mammalian cell damage inflicted, for example, by *C. albicans* and *Rhizopus delemar* (44, 45). However, the use of radioactive materials comes with their own dangers and difficulties and requires specialized training and facilities that are not easily accessible. This makes Nluc-reporter cell lines an attractive alternative for assaying host-cell-specific damage.

Luciferase-expressing cells have also been recently used to quantify damage to transduced cells in models that contain a mixture of mammalian cells, for example, to quantify the cytotoxicity of chimeric antibody-expressing immune cells that kill luciferase-transduced cancer cells (16) and to screen antibodies from patient blood that mediate antibody-dependent cellular toxicity toward cancer cells (46). This approach has also been used to identify small molecules that modulate natural killer cells to kill luciferase-transduced cancer cells more readily (47). In this manuscript, we tested applications for epithelial-Nluc in 2D cell culture models. Historically, these models have provided molecular insights into commensal and pathogenic interactions of fungi with specific cell types (reviewed in reference 34). Finally, the Nluc-reporter cell lines can be used in organ-on-chip or trans-well models that include immune cells which are key players in host-pathogen interactions (48; reviewed in reference 34). Here, our luciferase-reporter cell lines would be able to distinguish between damage to immune and epithelial components.

To summarize, we developed a luciferase-based assay to detect cytotoxicity in mammalian epithelial cell lines. The assay is simple, reliable, inexpensive, and can be used to detect even low levels of cell damage. It is also more specific than the traditional lactate dehydrogenase (LDH) assay, as it can distinguish between damage to transduced cells and damage to other cells in the culture. We tested the Nluc-reporter cell lines extensively in *C. albicans* infection model and found that the assay could detect the damage caused by the pathogen and that it was comparable to or even better than the LDH assay. While *C. albicans* served as a good model and case study, our data show that the assay should be applicable with minimal changes to test bacterial, viral, or protozoan infections as well as a range of cell-damaging substances. We also tested the stability of the assay over a range of temperatures, storage durations, and pH. We found that the assay was stable under a wide range of conditions, making it suitable for a variety of applications. Overall, our results suggest that the luciferase-based assay is a promising new tool for the detection of cytotoxicity in mammalian epithelial cell lines.

## MATERIALS AND METHODS

### *Candida* strains and cultivation

*Candida* sp. strains (Table 1), cryopreserved at −80°C, were streaked onto YPD-agar plates and incubated at 37°C for 24 h. Overnight cultures were prepared by inoculating single colonies into YPD 1% peptone in Erlenmeyer flasks for incubation at 30°C, 180 rpm.

**TABLE 1:** Candida strains used in this study

Species	Strain name	Genotype	Internal ID	Reference
*C. albicans*	SC5314	Genome sequence reference strain	C55	(49)
	BWP17 + CIp30	BWP17, *rps1*::(*HIS1 ARG4 URA3*)	M130	(50)
	101	Oral isolate from healthy volunteer, kindly provided by S. LeibundGut-Landmann	C222	(18)
*C. glabrata*	ATCC 2001	Genome sequence reference strain	C94	(51)
*C. parapsilosis*	GA1	*C. parapsilosis* WT strain GA1	C118	(52)
*C. tropicalis*	DSM 4959	*C. tropicalis* WT strain DSM 4959	C30	(53)
*C. albicans*	*efg1*ΔΔ/ *cph1*ΔΔ	SC5314, *cph1*::FRT1/*cph1*::FRT1 *efg1*::FRT/*efg1*::FRT	M2188	(19)
	*eed1*ΔΔ	SC5314, *eed1*::FRT/*eed1*::(FRT-SAT1-FRT)	M1314	(54)
	*bud2*ΔΔ	BWP17, *bud2*::*ARG4*/p*URA3-BUD2*::*bud2*::*HIS1*	M1411	(22)
	*ece1-III*ΔΔ	BWP17, *ece1*::*HIS1*/*ece1*::*ARG4* rps1::(*URA3 ECE1*^ΔPIII^)	M2174	(7)

### Molecular biology, lentiviral transfection, and transduction

The Nluc gene was amplified with pCRISPaint-NanoLuc plasmid purchased from Addgene (Veit Hornung Addgene plasmid #67178) and integrated with an IRES sequence (Veit Hornung Addgene plasmid #128061) and the puromycin resistance gene “PuroR” (Veit Hornung Addgene plasmid #78908) into the plasmid back bone of the vector “pHRSIN-cPPTSE,” also called “pSEW” (55). The insertion was confirmed by PCR (primers pSEW_BamHI-Nluc fw, GCCCGGGGGGGATCCGCCGCCACCATGGTCTTCACACTCGAAGATTTCG and Nluc-BamHI-IRES rev, AGAGGGGCGGGATCCTTACGCCAGAATGCGTTCGCACAGCCGCC).

For generation of lentivirus-like particles, HEK-293T/17 cells were transfected with a mixture of the transfer plasmid pSEW-NanoLuc-IRES-PuroR and helper plasmids psPAX2 (Addgene plasmid #12260) and pMD2G (Addgene plasmid #12259), combined in jetPRIME transfection agent buffer, as per manufacturer’s protocol. Lentiviral supernatant was filtered and concentrated with the Lenti-X concentrator as per the manufacturer’s protocol (Clontech Labs). A431 and TR146 cells were transduced by infecting cells with lentiviral particles for 48 h and selecting them in puromycin selection medium for 10–14 days. Selected cells were passaged and cryo-preserved in 10% DMSO + heat inactivated fetal bovine serum (FBS).

### Mammalian cell lines and cell culture

Mammalian cells were cultured and passaged in their respective medium (Table 2), supplemented with 10% FBS in 75 cm² tissue culture flasks. Confluent TR146 and A431 cells were detached using Accutase for 15–20 min. Detached cells were resuspended in three times the volume of their respective medium supplemented with 10% FBS (Table 2) and counted using a Neubauer counting chamber. A cell suspension containing 10^5^ cells/mL was prepared, and 200 µl of cell suspension was distributed evenly into a 96-well plate. Cells were incubated at 37°C, 5% CO_2_ for ≈2 days.

**TABLE 2:** Mammalian cell culture

Cell line name	Cell line description	Genotype	Medium used	Reference
TR146	Human buccal (oral) epithelial cell line	Wild type	DMEM:Ham’s F12 medium (1:1)	ECACC (from Sigma Aldrich), Lot No. 13D016
A431	Human vaginal epithelial cell line	Wild type	RPMI 2 g/L glucose	DSMZ

### Western blot to confirm the stability of luciferase expression

To confirm the stability of Nluc expression across passages of transduced cells, cells from consecutive passages were collected and centrifuged at 10,000 × *g,* and the pellet stored at −20°C. Cells were lysed in Radio-Immunoprecipitation Assay (RIPA) buffer (Merck) with protease inhibitor cocktail (Calbiochem) and Benzonase (25 U/µL) (Novagen). Proteins were quantified by bicinchoninic acid assay and run on an SDS PAGE (12% separating, 5% stacking) in Laemmli buffer. Protein bands were transferred to nitrocellulose membranes and incubated with monoclonal anti-Nanoluc antibody (Promega) and monoclonal anti-beta Tubulin antibody (AbD Serotec). Proteins were visualized using anti-mouse HRP-conjugated secondary antibody (Pierce) with an Odyssey M imaging system (LI-COR Biosciences), and images were processed in Image Studio (LI-COR Biosciences).

### Epithelial cell damage assays

To measure damage from cells seeded in 96-well plates, plates were centrifuged at 250 × *g* for 10 min. Where indicated, 100 µL of supernatant was collected and stored at −20°C for assaying on another day in the same week. For the luciferase assays, 5 µL supernatant was diluted with 95 µL PBS in white flat-opaque-bottomed 96-well plates (Thermoficher). Then 100 µL of 10 µM coelenterazine (in PBS) was added and luciferase activity was measured immediately using a Tecan infinite M200 luminescence reader, with an integration time of 100 ms and OD2 compensation. The OD2 neutral density filter attenuates high-level light by a factor of 100.

The LDH cytotoxicity assay was performed according to instructions in the Roche Cytotoxicity Detection Kit with minor modifications. Briefly, 20 µL supernatant was diluted with 80 µL PBS in transparent-flat-bottomed 96-well plates, 100 µL of kit reagent mixture was added to each well, the plates were incubated in the dark for 15–20 min at room temperatures, and absorbance was measured with a Tecan absorbance reader at 492 nm (reference at 600 nm).

### Quantifying Nluc activity per TR146-Nluc cell from lysate

TR146-Nluc cells were detached with Accutase, resuspended in PBS, and counted with a Neubauer counting chamber. A dilution of 2 × 10^6^ cells/mL was prepared in PBS, which was serially diluted 1:10 till 2 × 1^10^ cells/mL in lysis buffer (1% Triton X-100 in PBS), and incubated at 37°C for 30 min. One hundred microliters of 10 µM coelenterazine was added to 100 µL of cell lysate, such that we measured Nluc activity ranging from ≈1 to ≈10^5^ cells.

### Infection of epithelial cells with *Candida* sp. strains

Overnight cultures of *Candida* sp. strains in YPD, 1% peptone were diluted, washed in PBS, and counted using Neubauer counting chambers. The medium of confluent epithelial cells was replaced with a suspension of 1 × 10^5^ cells/mL *Candida* spp. (MOI 1) in the appropriate cell culture medium (without FBS). The cells were incubated at 37°C and 5% CO2 for 24 h.

### Time course of damage to epithelial cells induced by *C. albicans*

TR146-Nluc cells were seeded into 96-well plates and infected with *C. albicans* (SC5314) at MOI 1, as per the protocol detailed above, and supernatants were collected from separate wells at 0, 3, 8, 12, and 24 h and stored at −20°C. Samples were thawed the next day, centrifuged (250 × *g*), and assayed for luciferase and LDH activity.

### Infection of epithelial cells with *C. albicans* in the presence of antifungals

Stock solutions of the antifungal drugs, fluconazole (10 mg/mL), and voriconazole (10 mg/mL) were prepared in DMSO and stored at −20°C. A twofold dilution series was prepared in DMEM/F12 cell culture medium for fluconazole (8–0.25 µg/mL) and voriconazole (1–0.3125 µg/mL). Diluted drugs were transferred to TR146-Nluc cells seeded 48 h prior and infected with *C. albicans* (SC5314) as described above. Supernatant was collected 24 h after infection to measure damage by LDH and Nluc.

### *L. rhamnosus* colonization and *C. albicans* infection *in vitro* vaginal epithelial model

*L. rhamnosus* ATCC 7469 was cultured in MRS Broth for 48 h at 37°C with 5% CO_2_ and 1% O_2_. *L. rhamnosus* was washed in PBS and diluted in RPMI-1640 to optical density 0.2 (OD_600_). For *L. rhamnosus* colonization, 16–18 h prior to *C. albicans* infection, the medium of A431-Nluc was replaced with 50 µL of a suspension of *L. rhamnosus* in RPMI-1640. For fungal infection, 50 µL of *C. albicans* suspension (MOI 1) was prepared in RPMI-1640 and added to the colonized cells. After 24 h, supernatant was collected for luciferase and LDH assays.

### Robustness of Nluc and LDH activity at different pHs, temperatures, and pyruvate concentrations

TR14-Nluc cells were detached with Accutase and resuspended in 5 mL PBS. Cells were counted and ≈1 × 10^4^ cells/mL were resuspended in lysing buffer (1% Triton X-100 in PBS, pH 7.4).

For the effect of different pH on the enzyme activities, the lysis buffer was adjusted to pH 3, 4, 5, 7, 8, 9, 10, or 11 with HCl or NaOH, and this buffer was used to resuspend cells, which were subsequently incubated for 10 min at 37°C and centrifuged at 10,000 *× g* to remove cell debris, and supernatant was collected to measure LDH and luciferase activity.

To test the effect of storage at different temperatures over time, the cell lysates were incubated for 10 min at 37°C and centrifuged at 10,000 *× g*, and supernatant aliquots were stored at 37°C, room temperature (≈28° C), 8°C, or −20°C. One aliquot was thawed every other day to measure luciferase and LDH activity.

To examine the effects of pyruvate on the activity of LDH, a twofold dilution series for sodium pyruvate (16–0.5 mM) and lactate (0–60 mM) was prepared in our lysis buffer, and the lysate was incubated for 10 min at 37°C and centrifuged at 10,000 *× g*. The supernatant was collected to test LDH and luciferase enzyme activity.

## SUPPLEMENTAL MATERIAL

Figure S1 msphere.00509-24-s0001.pdf Western blot to confirm stability of Nluc expression across different passages.

ASM does not own the copyrights to Supplemental Material that may be linked to, or accessed through, an article. The authors have granted ASM a non-exclusive, world-wide license to publish the Supplemental Material files. Please contact the corresponding author directly for reuse.

## References

[1] Niles AL, Moravec RA, Riss TL. 2008. Update on in vitro cytotoxicity assays for drug development. Expert Opin Drug Discov 3:655–669. doi:10.1517/17460441.3.6.655 23506147

[2] Riss T, Niles A, Moravec R, Karassina N, Vidugiriene J. 2019. Cytotoxicity assays: *in vitro* methods to measure dead cells. In Assay guidance manual. Eli Lilly & Company and the National Center for Advancing Translational Sciences. 31070879

[3] Rodrigues ML, Nosanchuk JD. 2020. Fungal diseases as neglected pathogens: a wake-up call to public health officials. PLoS Negl Trop Dis 14:e0007964. doi:10.1371/journal.pntd.0007964 32078635 PMC7032689

[4] Brown GD, Denning DW, Gow NAR, Levitz SM, Netea MG, White TC. 2012. Hidden killers: human fungal infections. Sci Transl Med 4:165rv13. doi:10.1126/scitranslmed.3004404 23253612

[5] Brunke S, Mogavero S, Kasper L, Hube B. 2016. Virulence factors in fungal pathogens of man. Curr Opin Microbiol 32:89–95. doi:10.1016/j.mib.2016.05.010 27257746

[6] Allert S, Förster TM, Svensson C-M, Richardson JP, Pawlik T, Hebecker B, Rudolphi S, Juraschitz M, Schaller M, Blagojevic M, Morschhäuser J, Figge MT, Jacobsen ID, Naglik JR, Kasper L, Mogavero S, Hube B. 2018. Candida albicans-induced epithelial damage mediates translocation through intestinal barriers. MBio 9:e00915-18. doi:10.1128/mBio.00915-18 29871918 PMC5989070

[7] Moyes DL, Wilson D, Richardson JP, Mogavero S, Tang SX, Wernecke J, Höfs S, Gratacap RL, Robbins J, Runglall M, et al.. 2016. Candidalysin is a fungal peptide toxin critical for mucosal infection. Nature New Biol 532:64–68. doi:10.1038/nature17625 PMC4851236 27027296

[8] Sanchez AA, Johnston DA, Myers C, Edwards JE, Mitchell AP, Filler SG. 2004. Relationship between Candida albicans virulence during experimental hematogenously disseminated infection and endothelial cell damage in vitro. Infect Immun 72:598–601. doi:10.1128/IAI.72.1.598-601.2004 14688143 PMC344013

[9] Yamamura M, Boler J, Valdimarsson H. 1976. A chromium release assay for phagocytic killing of Candida albicans. J Immunol Methods 13:227–233. doi:10.1016/0022-1759(76)90069-7 796386

[10] Case NT, Duah K, Larsen B, Wong CJ, Gingras A-C, O’Meara TR, Robbins N, Veri AO, Whitesell L, Cowen LE. 2021. The macrophage-derived protein PTMA induces filamentation of the human fungal pathogen Candida albicans. Cell Rep 36:109584. doi:10.1016/j.celrep.2021.109584 34433036 PMC8454912

[11] Naglik JR, Gaffen SL, Hube B. 2019. Candidalysin: discovery and function in Candida albicans infections. Curr Opin Microbiol 52:100–109. doi:10.1016/j.mib.2019.06.002 31288097 PMC6687503

[12] Dubois R. 1885. Note sur la fonction photogenique chez les Pholades. Impr. G. Rougier.

[13] Fraga H. 2008. Firefly luminescence: a historical perspective and recent developments. Photochem Photobiol Sci 7:146–158. doi:10.1039/b719181b 18264582

[14] Baki A, Bielik A, Molnár L, Szendrei G, Keserü GM. 2007. A high throughput luminescent assay for glycogen synthase kinase-3beta inhibitors. Assay Drug Dev Technol 5:75–83. doi:10.1089/adt.2006.029 17355201

[15] Cox MC, Mendes R, Silva F, Mendes TF, Zelaya-Lazo A, Halwachs K, Purkal JJ, Isidro IA, Félix A, Boghaert ER, Brito C. 2021. Application of LDH assay for therapeutic efficacy evaluation of ex vivo tumor models. Sci Rep 11:18571. doi:10.1038/s41598-021-97894-0 34535719 PMC8448883

[16] Matta H, Gopalakrishnan R, Choi S, Prakash R, Natarajan V, Prins R, Gong S, Chitnis SD, Kahn M, Han X, Chaudhary V, Soni A, Sernas J, Khan P, Wang D, Chaudhary PM. 2018. Development and characterization of a novel luciferase based cytotoxicity assay. Sci Rep 8:199. doi:10.1038/s41598-017-18606-1 29317736 PMC5760659

[17] England CG, Ehlerding EB, Cai W. 2016. NanoLuc: a small luciferase is brightening up the field of bioluminescence. Bioconjug Chem 27:1175–1187. doi:10.1021/acs.bioconjchem.6b00112 27045664 PMC4871753

[18] Schönherr FA, Sparber F, Kirchner FR, Guiducci E, Trautwein-Weidner K, Gladiator A, Sertour N, Hetzel U, Le GTT, Pavelka N, d’Enfert C, Bougnoux M-E, Corti CF, LeibundGut-Landmann S. 2017. The intraspecies diversity of C. albicans triggers qualitatively and temporally distinct host responses that determine the balance between commensalism and pathogenicity. Mucosal Immunol 10:1335–1350. doi:10.1038/mi.2017.2 28176789

[19] Lo HJ, Köhler JR, DiDomenico B, Loebenberg D, Cacciapuoti A, Fink GR. 1997. Nonfilamentous C. albicans mutants are avirulent. Cell 90:939–949. doi:10.1016/s0092-8674(00)80358-x 9298905

[20] Martin R, Moran GP, Jacobsen ID, Heyken A, Domey J, Sullivan DJ, Kurzai O, Hube B. 2011. The Candida albicans-specific gene EED1 encodes a key regulator of hyphal extension. PLoS One 6:e18394. doi:10.1371/journal.pone.0018394 21512583 PMC3075580

[21] Riggle PJ, Andrutis KA, Chen X, Tzipori SR, Kumamoto CA. 1999. Invasive lesions containing filamentous forms produced by a Candida albicans mutant that is defective in filamentous growth in culture. Infect Immun 67:3649–3652. doi:10.1128/IAI.67.7.3649-3652.1999 10377153 PMC116558

[22] Hausauer DL, Gerami-Nejad M, Kistler-Anderson C, Gale CA. 2005. Hyphal guidance and invasive growth in Candida albicans require the Ras-like GTPase Rsr1p and its GTPase-activating protein Bud2p. Eukaryot Cell 4:1273–1286. doi:10.1128/EC.4.7.1273-1286.2005 16002653 PMC1168968

[23] Alonso-Roman R, Last A, Mirhakkak MH, Sprague JL, Möller L, Großmann P, Graf K, Gratz R, Mogavero S, Vylkova S, Panagiotou G, Schäuble S, Hube B, Gresnigt MS. 2022. Lactobacillus rhamnosus colonisation antagonizes Candida albicans by forcing metabolic adaptations that compromise pathogenicity. Nat Commun 13:3192. doi:10.1038/s41467-022-30661-5 35680868 PMC9184479

[24] Graf K, Last A, Gratz R, Allert S, Linde S, Westermann M, Gröger M, Mosig AS, Gresnigt MS, Hube B. 2019. Keeping Candida commensal: how lactobacilli antagonize pathogenicity of Candida albicans in an in vitro gut model. Dis Model Mech 12:dmm039719. doi:10.1242/dmm.039719 31413153 PMC6765188

[25] Gay RJ, McComb RB, Bowers GN. 1968. Optimum reaction conditions for human lactate dehydrogenase isoenzymes as they affect total lactate dehydrogenase activity. Clin Chem 14:740–753. 4299285

[26] Pekmezovic M, Kaune A-K, Austermeier S, Hitzler SUJ, Mogavero S, Hovhannisyan H, Gabaldón T, Gresnigt MS, Hube B. 2021. Human albumin enhances the pathogenic potential of Candida glabrata on vaginal epithelial cells. PLoS Pathog 17:e1010037. doi:10.1371/journal.ppat.1010037 34710198 PMC8577789

[27] Pfaller MA, Diekema DJ, Turnidge JD, Castanheira M, Jones RN. 2019. Twenty years of the SENTRY antifungal surveillance program: results for Candida species from 1997-2016. Open Forum Infect Dis 6:S79–S94. doi:10.1093/ofid/ofy358 30895218 PMC6419901

[28] Ademe M, Girma F. 2020. Candida auris: from multidrug resistance to pan-resistant strains. Infect Drug Resist 13:1287–1294. doi:10.2147/IDR.S249864 32440165 PMC7211321

[29] Li D, She X, Calderone R. 2020. The antifungal pipeline: the need is established. Are there new compounds? FEMS Yeast Res 20:foaa023. doi:10.1093/femsyr/foaa023 32353872

[30] Roemer T, Krysan DJ. 2014. Antifungal drug development: challenges, unmet clinical needs, and new approaches. Cold Spring Harb Perspect Med 4:a019703. doi:10.1101/cshperspect.a019703 24789878 PMC3996373

[31] Finkel JS, Mitchell AP. 2011. Genetic control of Candida albicans biofilm development. Nat Rev Microbiol 9:109–118. doi:10.1038/nrmicro2475 21189476 PMC3891587

[32] Homann OR, Dea J, Noble SM, Johnson AD. 2009. A phenotypic profile of the Candida albicans regulatory network. PLoS Genet 5:e1000783. doi:10.1371/journal.pgen.1000783 20041210 PMC2790342

[33] Noble SM, French S, Kohn LA, Chen V, Johnson AD. 2010. Systematic screens of a Candida albicans homozygous deletion library decouple morphogenetic switching and pathogenicity. Nat Genet 42:590–598. doi:10.1038/ng.605 20543849 PMC2893244

[34] Last A, Maurer M, S Mosig A, S Gresnigt M, Hube B. 2021. In vitro infection models to study fungal-host interactions. FEMS Microbiol Rev 45:fuab005. doi:10.1093/femsre/fuab005 33524102 PMC8498566

[35] Cowen LE, Sanglard D, Howard SJ, Rogers PD, Perlin DS. 2014. Mechanisms of antifungal drug resistance. Cold Spring Harb Perspect Med 5:a019752. doi:10.1101/cshperspect.a019752 25384768 PMC4484955

[36] Van den Bossche S, Vandeplassche E, Ostyn L, Coenye T, Crabbé A. 2020. Bacterial interference with lactate dehydrogenase assay leads to an underestimation of cytotoxicity. Front Cell Infect Microbiol 10:494. doi:10.3389/fcimb.2020.00494 33042868 PMC7523407

[37] Hall MP, Unch J, Binkowski BF, Valley MP, Butler BL, Wood MG, Otto P, Zimmerman K, Vidugiris G, Machleidt T, Robers MB, Benink HA, Eggers CT, Slater MR, Meisenheimer PL, Klaubert DH, Fan F, Encell LP, Wood KV. 2012. Engineered luciferase reporter from a deep sea shrimp utilizing a novel imidazopyrazinone substrate. ACS Chem Biol 7:1848–1857. doi:10.1021/cb3002478 22894855 PMC3501149

[38] Lindén SK, Driessen KM, McGuckin MA. 2007. Improved in vitro model systems for gastrointestinal infection by choice of cell line, pH, microaerobic conditions, and optimization of culture conditions. Helicobacter 12:341–353. doi:10.1111/j.1523-5378.2007.00509.x 17669108

[39] Gleason FH, Nolan RA, Wilson AC, Emerson R. 1966. D(—)-lactate dehydrogenase in lower fungi. Science 152:1272–1273. doi:10.1126/science.152.3726.1272 4287062

[40] Lockwood LB, Ward GE. 1936. The physiology of Rhizopus oryzae. J Agric Res 53

[41] Garvie EI. 1980. Bacterial lactate dehydrogenases. Microbiol Rev 44:106–139. doi:10.1128/mr.44.1.106-139.1980 6997721 PMC373236

[42] Dunn CR, Banfield MJ, Barker JJ, Higham CW, Moreton KM, Turgut-Balik D, Brady RL, Holbrook JJ. 1996. The structure of lactate dehydrogenase from Plasmodium falciparum reveals a new target for anti-malarial design. Nat Struct Biol 3:912–915. doi:10.1038/nsb1196-912 8901865

[43] Makler MT, Piper RC, Milhous WK. 1998. Lactate dehydrogenase and the diagnosis of malaria. Parasitol Today 14:376–377. doi:10.1016/s0169-4758(98)01284-8 17040820

[44] Fukuoka T, Johnston DA, Winslow CA, de Groot MJ, Burt C, Hitchcock CA, Filler SG. 2003. Genetic basis for differential activities of fluconazole and voriconazole against Candida krusei. Antimicrob Agents Chemother 47:1213–1219. doi:10.1128/AAC.47.4.1213-1219.2003 12654649 PMC152512

[45] Soliman SSM, Baldin C, Gu Y, Singh S, Gebremariam T, Swidergall M, Alqarihi A, Youssef EG, Alkhazraji S, Pikoulas A, Perske C, Venkataramani V, Rich A, Bruno VM, Hotopp JD, Mantis NJ, Edwards JE Jr, Filler SG, Chamilos G, Vitetta ES, Ibrahim AS. 2021. Mucoricin is a ricin-like toxin that is critical for the pathogenesis of mucormycosis. Nat Microbiol 6:313–326. doi:10.1038/s41564-020-00837-0 33462434 PMC7914224

[46] Vincken R, Ruiz-Saenz A. 2023. A co-culture model system to quantify antibody-dependent cellular cytotoxicity in human breast cancer cells using an engineered natural killer cell line. STAR Protoc 4:102224. doi:10.1016/j.xpro.2023.102224 37071532 PMC10323021

[47] Cortés-Kaplan S, Kurdieh R, Hasim MS, Kaczmarek S, Taha Z, Maznyi G, McComb S, Lee S-H, Diallo J-S, Ardolino M. 2022. A new functional screening platform identifies colistin sulfate as an enhancer of natural killer cell cytotoxicity. Cancers (Basel) 14:2832. doi:10.3390/cancers14122832 35740500 PMC9221353

[48] Alonso-Roman R, Mosig AS, Figge MT, Papenfort K, Eggeling C, Schacher FH, Hube B, Gresnigt MS. 2024. Organ-on-chip models for infectious disease research. Nat Microbiol 9:891–904. doi:10.1038/s41564-024-01645-6 38528150

[49] Jones T, Federspiel NA, Chibana H, Dungan J, Kalman S, Magee BB, Newport G, Thorstenson YR, Agabian N, Magee PT, Davis RW, Scherer S. 2004. The diploid genome sequence of Candida albicans. Proc Natl Acad Sci U S A 101:7329–7334. doi:10.1073/pnas.0401648101 15123810 PMC409918

[50] Fonzi WA, Irwin MY. 1993. Isogenic strain construction and gene mapping in Candida albicans. Genetics 134:717–728. doi:10.1093/genetics/134.3.717 8349105 PMC1205510

[51] Dujon B, Sherman D, Fischer G, Durrens P, Casaregola S, Lafontaine I, De Montigny J, Marck C, Neuvéglise C, Talla E, et al.. 2004. Genome evolution in yeasts. Nat New Biol 430:35–44. doi:10.1038/nature02579 15229592

[52] Gácser A, Trofa D, Schäfer W, Nosanchuk JD. 2007. Targeted gene deletion in Candida parapsilosis demonstrates the role of secreted lipase in virulence. J Clin Invest 117:3049–3058. doi:10.1172/JCI32294 17853941 PMC1974868

[53] Borg M, Kirk D, Baumgarten H, Rüchel R. 1984. A colorimetric assay for the assessment of cytotoxicity of yeasts. Sabouraudia 22:357–367. doi:10.1080/00362178485380611 6505912

[54] Zakikhany K, Naglik JR, Schmidt-Westhausen A, Holland G, Schaller M, Hube B. 2007. In vivo transcript profiling of Candida albicans identifies a gene essential for interepithelial dissemination. Cell Microbiol 9:2938–2954. doi:10.1111/j.1462-5822.2007.01009.x 17645752

[55] Mock U, Riecken K, Berdien B, Qasim W, Chan E, Cathomen T, Fehse B. 2014. Novel lentiviral vectors with mutated reverse transcriptase for mRNA delivery of TALE nucleases. Sci Rep 4:6409. doi:10.1038/srep06409 25230987 PMC4166709

