# Supplementary material for: Shining a light on Candida-induced epithelial damage with a luciferase reporter
Source: mSphere. 2024 Oct 16;9(11):e00509-24. doi: 10.1128/msphere.00509-24 (PMC11580449; doi:10.1128/msphere.00509-24)
Supplement: Figure S1 — Western blot to confirm stability of Nluc expression across different passages. [file msphere.00509-24-s0001.pdf]

A

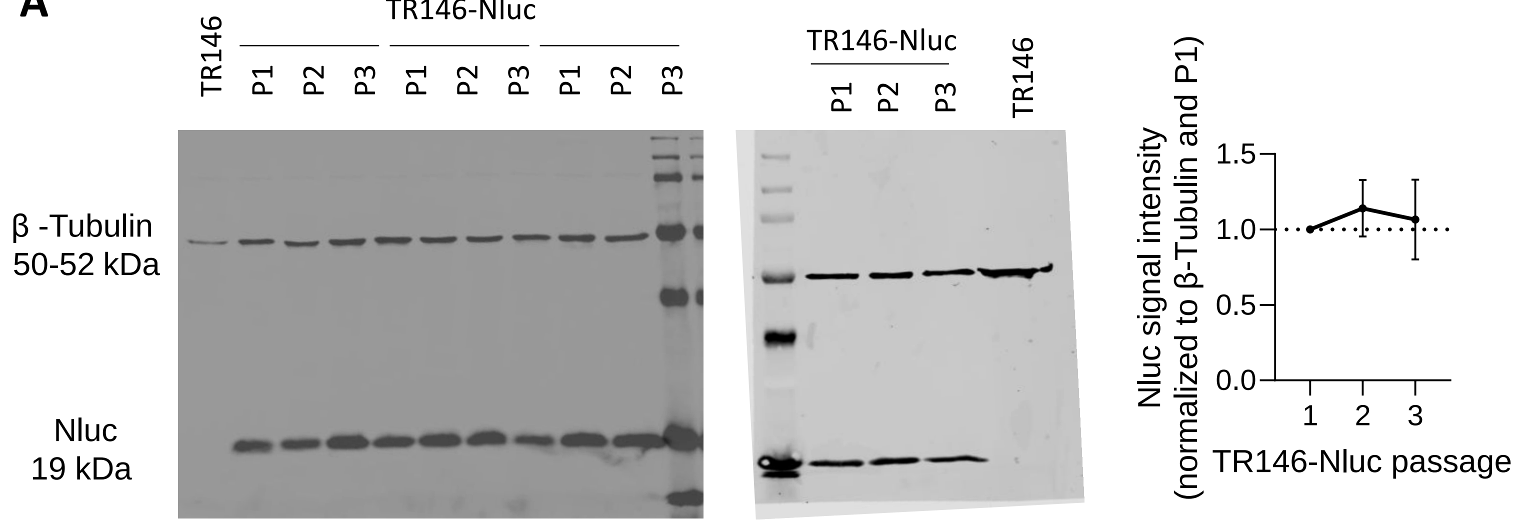

B

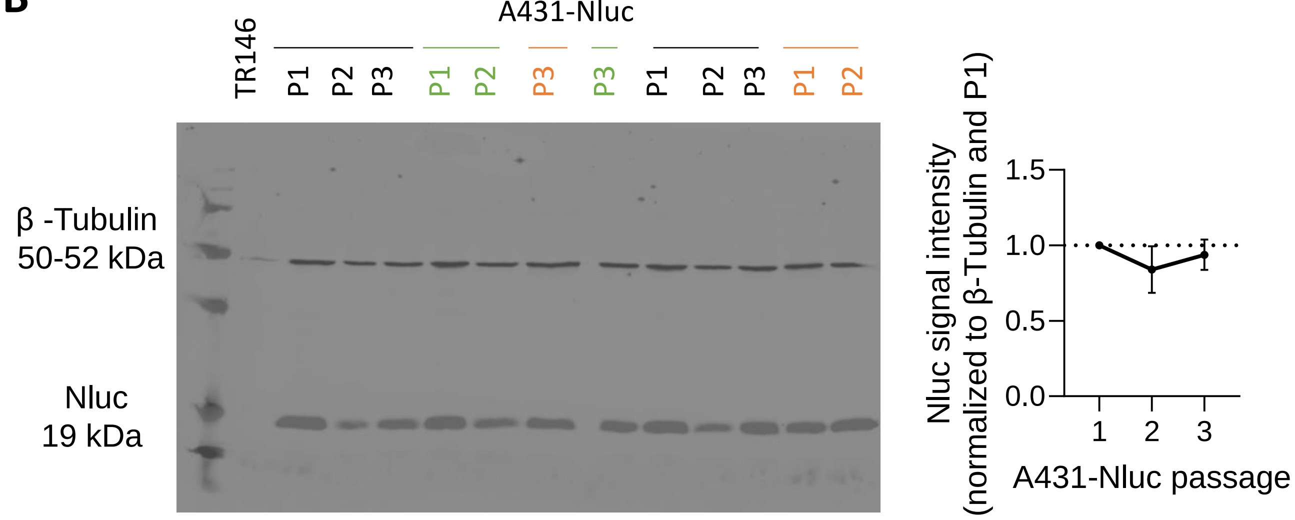

Figure S1: Cell lysates from **A.** TR146-Nluc and **B.** A431-Nluc were collected over three passages, solubilized for SDS-PAGE, blotted and proteins detected with the indicated antibodies. Representative image of four independent replicates shows that TR146-Nluc and A431-Nluc cells stably expressing Nluc. The stability of Nluc expression in TR146-Nluc and A431-Nluc is normalized to the loading control β-Tubulin, relative to an arbitrary passage (P1, dotted line), was quantified using image J, and is depicted in panels to the right of the respective blot. The error-line represents the standard devation about the mean (black point).
